# Hyperuricemia and smoking in young adults suspected of coronary artery disease ≤ 35 years of age: a hospital-based observational study

**DOI:** 10.1186/s12872-018-0910-5

**Published:** 2018-08-31

**Authors:** Sai Lv, Wei Liu, Yujie Zhou, Yuyang Liu, Dongmei Shi, Yingxin Zhao, Xiaoli Liu

**Affiliations:** Department of Cardiology, Beijing Anzhen Hospital, Capital Medical University, Beijing Institute of Heart Lung and Blood Vessel Disease, Anzhen Ave #2, Chaoyang District, Beijing, 100029 China

**Keywords:** Coronary artery disease, Hyperuricemia, Cigarette smoking, Young adults

## Abstract

**Background:**

Coronary artery disease (CAD) is showing an increasing trend in young adults. Cigarette smoking has been shown to be a major cause of premature CAD. Previous studies have also shown that hyperuricemia (HUA) is associated with CAD; however, the interaction effect between HUA and smoking on CAD is uncertain. Therefore, this study was designed to determine the relationship and interactive effects of HUA and smoking on the risk of CAD in young adults ≤ 35 years of age.

**Methods:**

In this observational study we consecutively included adults (18–35 years of age) with suspected CAD who underwent coronary angiography for the first time in our institution from January 2005 to December 2015. Patients with stenosis affecting ≥50% of the luminal diameter and acute myocardial infarction were considered to have CAD. A serum uric acid (SUA) level ≥ 7.0 mg / dl (420 mmol / L) in males and ≥ 6.0 mg / dl (357 mmol / L) in females was defined as hyperuricemia. We tested for an interaction between HUA and cigarrete smoking on CAD. The relationship between HUA, cigarrete smoking, and CAD was assessed by multivariate logistic regression analysis.

**Results:**

A total of 1113 participants were included in this study; 771 participants were confirmed to have CAD. HUA was present in 34.8% of the participants. HUA was significantly higher in the CAD group (odds ratio [OR], 1.34; 95% confidence interval [CI], 1.02–1.76; *p* = 0.035). More smokers were in the CAD group (OR, 1.59; 95% CI, 1.22–2.07; *p* = 0.001). Based on multivariate regression analysis and after adjustment for age, BMI, high LDL-C level, low HDL-C level, hypercholesterolemia, hypertriglyceridemia, metabolic syndrome, diabetes mellitus, and hypertension, HUA was shown to be strongly associated with the presence of CAD in non-smokers (OR, 1.84; 95% CI, 1.03–3.29; *p* = 0.039). We further demonstrated that the interaction between HUA and cigarrete smoking achieved statistical significance for the presence of CAD (*p* = 0.008).

**Conclusions:**

In the current study, HUA was shown to be associated with the presence of CAD in non-smokers ≤ 35 years of age.

**Electronic supplementary material:**

The online version of this article (10.1186/s12872-018-0910-5) contains supplementary material, which is available to authorized users.

## Background

Coronary artery disease (CAD) is relatively rare in patients < 40 years of age, occurring in 6–10% of all patients < 40 years of age; however, there has recently been an increase in incidence [1, 2]. A variety of risk factors are known to be involved in the onset and progression of CAD [3], the contributions of which vary in patients of different ages. Cigarette smoking has been shown to be a major cause of premature CAD. Several studies have described the relationship between hyperuricemia (HUA) and CAD in middle-aged and elderly populations [4, 5]. These studies have shown that HUA may play a role in CAD; however, due to the relatively low prevalence of CAD in young adults, the association between HUA and CAD in young adults has not elicited attention among the public.

Given the oxidative and inflammatory-induced properties of HUA and cigarrete smoking, along with the associations with risk for CAD, investigating the effect of the interaction between HUA and current smokers on premature CAD is warranted.

Thus, the current study was designed to determine the following: 1) association between HUA and CAD; 2) association between cigarette smoking and CAD; 3) interactive effect of both factors on the risk for CAD in young adults ≤ 35 years of age; and 4) the association between HUA and cigarrete smoking in the study participants.

## Methods

### Study population

In this observational study, young adults suspected to have CAD (18–35 years of age) who underwent coronary angiography for the first time at Anzhen Hospital between January 2005 and December 2015 were consecutively enrolled. Our study was approved by the Institutional Ethics Committee at Beijing Anzhen Hospital. Written informed consent was gained from all participants. The exclusion criteria were as follows: 1. gout, heart failure, renal impairment (an estimated glomerular filtration rate [eGFR] < 60 mL/minute per 1.73 m^2^), inflammatory diseases, autoimmune diseases (Takayasu’s arteritis, infective endocarditis, and rheumatic heart disease), and missing uric acid data; 2. medication history prior to admission, including diuretics or anti-hypertension drugs, losartan and hydrochlorothiazide tablets, compound amiloride hydrochloride tablets, irbesartan, and hydrochlorothiazide, which are known to affect the level of uric acid; 3. previous percutaneous coronary intervention or coronary artery bypass grafting in our institution; and 4. congenital heart disease, cardiomyopathy, and valvular heart disease.

### Ascertainment of outcome

Coronary angiography was performed using a standardized technique. Coronary angiogram results were affirmed by two experienced cardiologists. Major epicardial coronary arteries, including the left main, left anterior descending, left circumflex, right coronary artery, and the vessels’ main branches, were evaluated, and a luminal diameter stenosis ≥50% in any of the vessels above was defined as CAD. Patients with a history of an acute myocardial infarction were also considered to have CAD.

### Definition and collection of variables

Based on published clinical guidelines, HUA was defined as a serum uric acid (SUA) level ≥ 7.0 mg/dl (420 mmol / L) in males and ≥ 6.0 mg / dl (357 mmol/L) in females [6]. Hypertension was defined as a blood pressure ≥ 140/90 mmHg or using anti-hypertension medications [7]. Diabetes mellitus was defined according to the WHO diabetes diagnostic criteria in 1999. LDL-C ≥ 130 mg/dl (3.4 mmol / L) was considered to be a high LDL-C level, TG ≥ 150 mg/dl (1.7 mmol / L) was considered to be hypertriglyceridemia, HDL-C < 40 mg/dl (1.0 mmol / L) was considered to be a low HDL-C level, and TC ≥ 200 mg/dl (5.2 mmol / L) was considered to be hypercholesterolemia [8].

SUA was analyzed on a Beckmann AU54 automated biochemical analyzer (Beijing, China) using a uric acid commercial kit (uricase-peroxidase method).

Personal histories of hypertension, diabetes mellitus, a family history of CAD, cigarette smoking, and alcohol consumption were collected from electronic medical records.

### Statistical analyses

Continuous variables are presented as the mean ± standard deviation and compared using an unpaired t-test for normally distributed data or as the median with interquartile range and compared using a Mann-Whitney U test for non-normally distributed data. Categorical variables are presented as frequencies or percentages and compared using a chi-square test. Univariate analysis was performed, then covariates with a *p* < 0.05 on the univariate analysis were analyzed for collinearity. The variables were then selected and added to a multivariable analysis. The relationship between HUA, cigarrete smoking, and the presence of CAD was evaluated with multivariate logistic regression analysis. A *p* value ≤0.05 (two-sided) was considered statistically significant. We tested for interactions between uric acid (HUA or normouricemia) and cigarrete smoking on the risk for CAD. A *p*-value for interaction was measured using the log likelihood ratio test to determine if there was a significant difference between the following two regression models: 1. a complex regression model including not only the exposure and confounding factors, but also the interaction terms of each of the factors with the effect modifier; and 2. a simple model without the interaction terms. With a *p*-value for interaction < 0.05, there was a significant difference between the two models, indicating that the complex model interpreted the data better. All analyses were performed with the statistical software package R and EmpowerStats (http://www.empowerstats.com; X&Y Solutions, Inc., Boston, MA, USA) [9].

## Results

### Patient demographics

A total of 1113 participants fulfilling the criteria were enrolled in this observational study. The mean age was 31.8 years and 93.7% of the participants were male. Additional file 1: Figure S1 shows the flow chart of the study. The 771 participants with ≥50% major cardiac artery narrowing and a history of an acute myocardial infarction made up the CAD group, and the remaining 342 participants comprised the non-CAD group. The baseline characteristics are shown in Table 1. Current smokers and hypertriglyceridemia were more prevalent in the CAD group than the non-CAD group (68.2% vs. 57.4% and 60.6% vs. 47.8%, respectively; *P*<0.001). The CAD group also had a decreased level of HDL-C (*P*<0.001). Moreover, a greater percentage of patients with HUA were in the CAD group (*p* = 0.034). In the CAD group, 21.6% of patients were treated with drugs.

**Table 1:** Baseline clinical characteristics in Non-CAD and CAD patients

Baseline Characteristics	Non-CAD group	CAD group	*p* value
	(*n* = 342)	(*n* = 771)	
Age (years)	32.1 ± 3.0	31.6 ± 3.4	0.033
Male, n (%)	318 (92.2)	730 (94.4)	0.149
Hyperuricemia, n (%)	104 (30.4)	285 (37.0)	0.034
Alcohol drinking, n (%)	86 (24.9)	194 (25.1)	0.952
BUN(mg/dL)	14.2 ± 7.8	12.4 ± 4.8	< 0.001
Serum creatinine(mg/dL)	0.9 ± 0.2	0.9 ± 0.2	0.278
Triglycerides(mg/dL)	145.3 (102.8–218.8)	172.8(118.1–250.5)	< 0.001
HDL-C(mg/dL)	38.1 ± 9.2	35.0 ± 8.0	< 0.001
LDL-C (mg/dL)	109.0 ± 32.3	114.7 ± 46.8	0.043
Total cholesterol (mg/dL)	173.4 ± 41.7	179.2 ± 55.5	0.084
Uric acid (mg/dL)	6.4 ± 1.6	6.6 ± 1.6	0.194
Fasting glucose (mg/dL)	96.8 ± 26.3	104.3 ± 33.0	< 0.001
BMI(kg/m^2^)	26.8 ± 4.2	27.9 ± 4.1	< 0.001
Traditional coronary risk factor, n (%)			
Current smokers, n (%)	198 (57.4)	527 (68.2)	< 0.001
Family history of CAD, n (%)	33 (9.6)	113 (14.6)	0.021
Hypertension, n (%)	135 (39.1)	324 (41.9)	0.382
Diabetes mellitus, n (%)	32 (9.3)	124 (16.0)	0.003
Metabolic syndrome, n (%)	168 (49.4)	450 (58.9)	0.003
Hypercholesterolemia, n (%)	74 (21.7)	208 (27.2)	0.055
Hypertriglyceridemia, n (%)	163 (47.8)	464 (60.6)	< 0.001
High LDL-C, n (%)	83 (24.3)	207 (27.0)	0.349
Low HDL-C, n (%)	235 (68.9)	586 (76.5)	0.008
familial hypercholesterolemia, n (%)	0 (0.0)	12 (1.6)	0.020
Treatment, n (%)			< 0.001
Drugs, n (%)	11 (3.2)	167 (21.6)	
Intervention, n (%)	0 (0.0)	528 (68.3)	
CABG, n (%)	0 (0.0)	77 (10.0)	
Aspirin, n (%)	162 (47.0)	742 (96.0)	< 0.001
Clopidogrel/ticagrelor, n (%)	48 (13.9)	639 (82.7)	< 0.001
Statin, n (%)	142 (41.2)	743 (96.1)	< 0.001
β-blocker, n (%)	103 (29.9)	630 (81.5)	< 0.001

Values are given as mean ± standard deviation,medians with interquartile range or number (%)

Abbreviations: *BUN* blood urea nitrogen, *HDL-C* high density lipoprotein cholesterol, *LDL-C* low density lipoprotein cholesterol, *BMI* body mass index, *CAD* coronary artery disease

### Univariate analysis of different CAD risk factors

Univariate analysis showed that the traditional CAD risk factors for cigarette smoking, BMI, family history of CAD, hypertriglyceridemia, low level of HDL-C, metabolic syndrome, and diabetes mellitus were significantly associated with the presence of CAD (P<0.05). In contrast, the risk factors for hypertension and hypercholesterolemia were not significantly related to CAD(Table 2). Within the entire patient group, univariate analysis revealed that HUA was significantly associated with the occurrence of CAD (OR, 1.34; 95% CI, 1.02–1.76; *p* = 0.035; Table 2).

**Table 2:** Univariate analysis of CAD risk factors

Variables	Mean ± SD	n (%)	Crude HR (95% CI)	*p* value
Age	31.8 ± 3.3		0.96 (0.92, 1.00)	0.034
Gender				
Male		1043 (93.7)	1.45 (0.88, 2.39)	0.144
Female		70 (6.3)	1.0	
Hyperuricemia				
Yes		389 (34.95)	1.34 (1.02, 1.76)	0.035
No		724 (65.05)	1.0	
BUN	12.9 ± 5.9		0.95 (0.93, 0.97)	< 0.001
Serum creatinine	0.9 ± 0.2		1.51 (0.72, 3.19)	0.278
BMI	27.6 ± 4.2		1.06 (1.03, 1.10)	< 0.001
Alcohol drinking				
Yes		280 (25.2)	1.00 (0.75, 1.34)	0.996
No		833 (74.8)	1.0	
Current smokers				
Yes		724 (65.0)	1.56 (1.20, 2.03)	< 0.001
No		389 (35.0)	1.0	
Family history of CAD				
Yes		146 (13.1)	1.61 (1.07, 2.42)	0.023
No		967 (86.9)	1.0	
Hypertension				
Yes		456 (41.0)	1.11 (0.86, 1.44)	0.419
No		657 (59.0)	1.0	
Diabetes mellitus				
Yes		156 (14.0)	1.86 (1.23, 2.80)	0.003
No		957 (86.0)	1.0	
Metabolic syndrome				
Yes		618 (56.0)	1.47 (1.13, 1.90)	0.003
No		486 (44.0)	1.0	
Total cholesterol				
TC > =200		282 (25.5)	1.34 (0.99, 1.82)	0.055
TC < 200		825 (74.5)	1.0	
Triglycerides				
TG > =150		627 (56.6)	1.68 (1.30, 2.17)	< 0.001
TG < 150		480 (43.4)	1.0	
LDL-C				
LDL-C > =130		290 (26.2)	1.15 (0.86, 1.54)	0.349
LDL-C < 130		817 (73.8)	1.0	
HDL-C				
HDL-C < 40		821 (74.2)	1.47 (1.11, 1.95)	0.008
HDL-C > =40		286 (25.8)	1.0	

Abbreviations: *BUN* blood urea nitrogen, *HDL-C* high density lipoprotein cholesterol, *LDL-C* low density lipoprotein cholesterol, *BMI* body mass index, *CAD* coronary artery disease

### Multivariate logistic regression analysis model of different traditional CAD risk factors

**Fig. 1:**
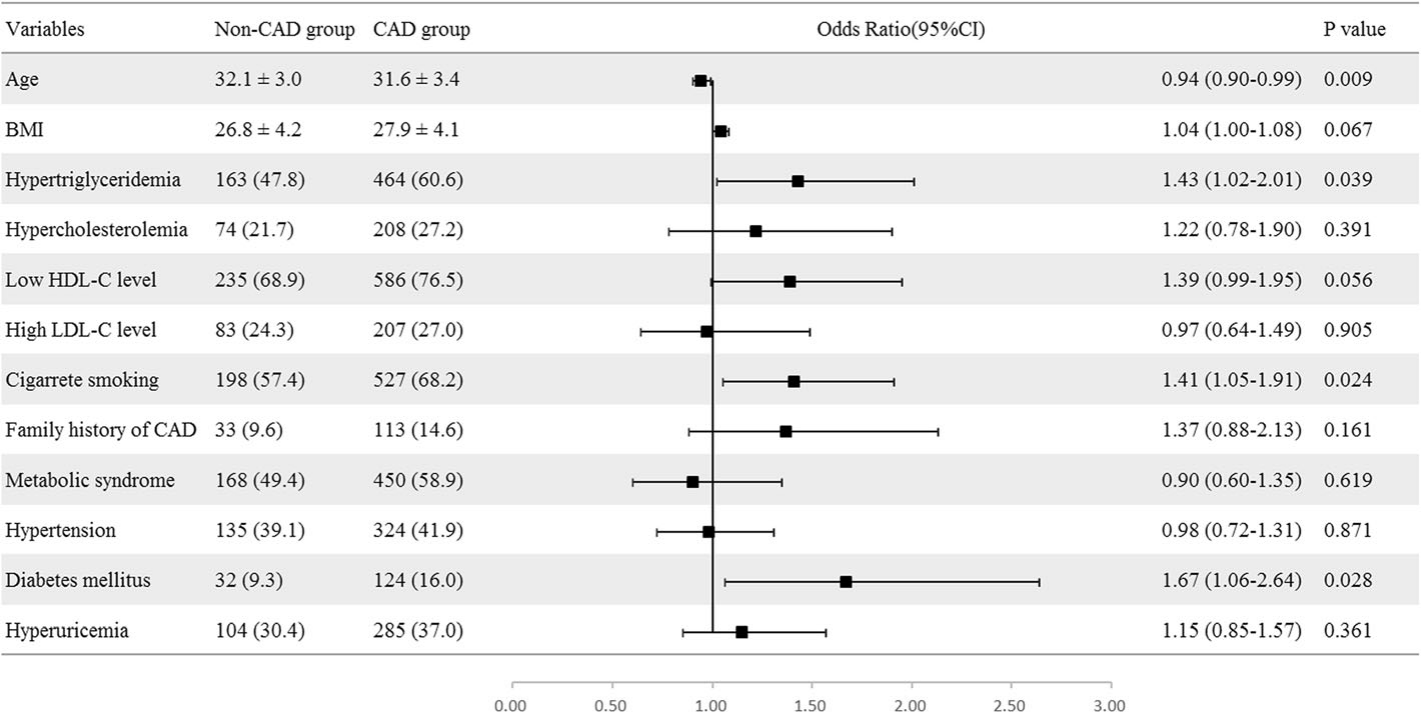
Forest plot of Multi-variate logistic regression analysis model of different CAD risk factors. Logistic regression analysis indicated that cigarrete smoking, diabetes mellitus and hypertriglyceridemia had significant associations with the presence of CAD. Hyperuricemia, BMI, family history of CAD, high LDL-C level, low HDL-C level, hypercholesterolemia and hypertension were not associated with the occurrence of CAD. The factors that were related to the dependent variables mentioned in previous studies enter into the univariate analysis. Covariates with *p* < 0.05 in the univariate analysis were analyzed for collinearity. Afterwards, the variables selected finally were added to the full models. Abbreviations: HDL-C = high density lipoprotein cholesterol; LDL-C = low density lipoprotein cholesterol; BMI = body mass index; CAD = coronary artery disease

Logistic regression analysis further indicated that the traditional CAD risk factors, such as cigarette smoking, diabetes mellitus, and hypertriglyceridemia, had significant associations with the presence of CAD (*P*<0.05; Fig. 1).

### Multivariate logistic regression analysis of HUA on CAD

**Fig. 2:**
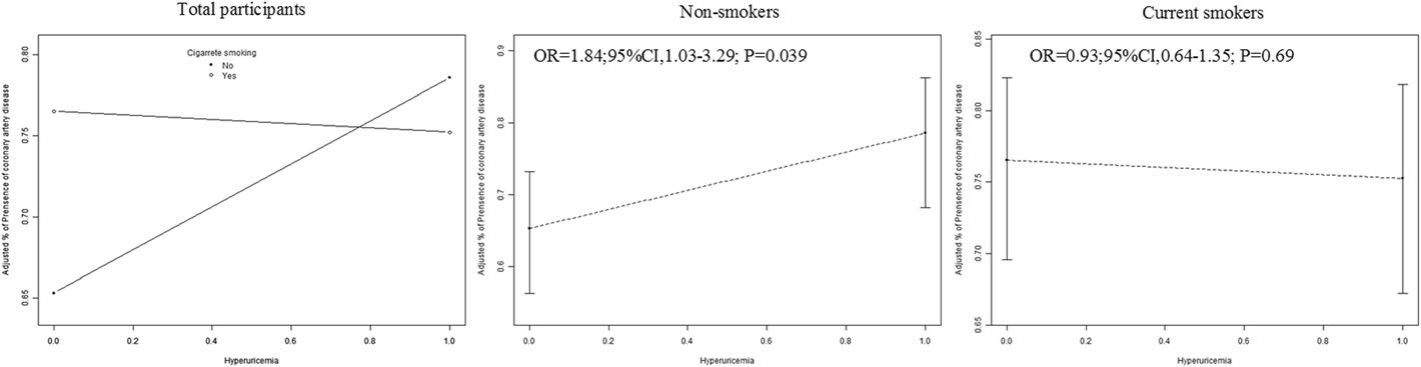
The association of hyperuricemia and the presence of CAD according to cigarrete smoking in Multi-variate logistic regression analysis. Abbreviations: CI = confidence interval; OR = odds ratio; CAD = coronary artery disease

In addition, the association between HUA and the occurrence of CAD differed between groups. Multivariate logistic analysis showed that after adjusting for age, high LDL-C, low HDL-C, hypercholesterolemia, hypertriglyceridemia, metabolic syndrome, BMI, hypertension, diabetes mellitus, and a family history of CAD, HUA was significantly associated with the occurrence of CAD in the subgroup of non-smokers (OR, 1.84; 95% CI, 1.03–3.29), but the association did not exist in the current smokers sub-group (OR, 0.93; 95% CI, 0.64–1.35; Table 3). Figure 2 shows the logistic association between HUA and the presence of CAD in the non-smoker (*p* = 0.039) and current smoker groups (*p* = 0.69).

**Table 3:** Association between hyperuricemia and CAD in different subgroups of smokers and non-smokers

Variables	No. of patients	Odds Ratio (95% CI)	
		Crude	Model I^a^
Non-Smokers			
Normouricemia	279	Reference	Reference
Hyperuricemia	110	2.08 (1.28, 3.40)	1.84 (1.03, 3.29)
Current smokers			
Normouricemia	445	Reference	Reference
Hyperuricemia	279	1.01 (0.72, 1.41)	0.93 (0.64, 1.35)

Abbreviations: *CAD* coronary artery disease, *HDL-C* high density lipoprotein cholesterol, *LDL-C* low density lipoprotein cholesterol, *BMI* body mass index, *CI* confidence interval, *OR* odds ratio

^a^Adjusted for Age, High LDL-C, Low HDL-C, Hypercholesterolemia, Hypertriglyceridemia, Metabolic syndrome, BMI, Hypertension, Diabetes mellitus, Family history of CAD, Blood urea nitrogen, Serum creatinine and Alcohol drinking

### Interaction effect of HUA and cigarrete smoking on the risk of CAD

We further found that the interaction between HUA and cigarrete smoking achieved statistical significance for the occurrence of CAD (*p* = 0.008; Additional file 1: Table S1).

### Relationship between HUA and cigarrete smoking in the study population

Moreover, we found more subjects with HUA among the current smokers (*p* < 0.001), and the current smoker group had higher levels of plasma uric acid than the non-smoker group (*p* < 0.001), both in males (*p* = 0.009) and females (*p* = 0.019; Additional file 1: Table S2).

## Discussion

### HUA and CAD

In this observational study, we confirmed that HUA was significantly associated with incident CAD in non-smokers ≤ 35 years of age.

Previous studies have also reported a relationship between HUA and CAD [4, 10–12]. Some of the results confirmed a strong association between HUA and CAD; however, most of the results focused on the relationship between HUA and the risk of CAD in a middle-aged and elderly population [11, 13]. The reports did not focus on the relationship between HUA and CAD in young adults. A study involving a population with an average age of 40 ± 4 years suggested that SUA levels were independently related to coronary calcifications (indicators of sub-clinical coronary atherosclerosis). The recent study concerning on the relationship between HUA and CAD in patients ≤35 years of age (the 2015 CARDIA study) showed that after adjusting for demographic and lifestyle factors, the baseline SUA concentration was positively associated with the prevalence of CVD (HR, 1.21; 95% CI, 1.05–1.39; *p* = 0.005). This positive association was not significant in the full model due to the simultaneous effects of traditional risk factors (HR, 1.09; 95% CI, 0.94–1.27; *p* = 0.24) [14]. This conclusion was similar to our findings. Compared with the above studies, our study had access to a wide range of covariates and minimized the residual confounding, and still demonstrated a strong association.

### Role of cigarette smoking

We stratified our patients into smokers versus non-smokers and found that the association between HUA and the presence of CAD existed in non-smokers only. In current smokers, we did not find a strong association between HUA and the presence of CAD, considering the stronger effect of cigarette smoking on young patients with CAD and the unbalanced gender ratio in the participants.

In addition, we identified more subjects with HUA among current smokers. The reasons for the increase in SUA levels in current smokers group may include the following: 1. Increasing SUA reabsorption. Studies have shown that smoking is positively correlated with the degree of insulin resistance, [15] and insulin resistance promotes sodium-hydrogen exchange in the renal proximal tubules [13]. With the increase in sodium-hydrogen exchange, anions, including uric acid, increase [16]. 2. Reducing the SUA excretion. Cigarettes contain a variety of harmful substances, including oxidants and free radicals that can result in a cytotoxic effect and lead to renal damage, thus eventually reducing the excretion of uric acid [17, 18]. A 2011 study of SUA and cigarette smoking among 300 volunteers 19.6–55.5 years of age showed a reduction in SUA levels in the smoking group [19]. These results were not consistent with our results, considering the differences in the age and the health status of the study participants, which need further study.

The current results can only explain the possible existence of the interaction between HUA and cigarette smoking on CAD, and the specific pathways need more in-depth research.

The clinical importance of this article is that for young adults, in addition to the main risk factors for premature CAD, such as cigarette smoking, non-traditional risk factors, such as HUA plays an important role in the occurrence of premature CAD. A young non-smoking population, from the perspective of coronary heart disease prevention, should pay more attention to the adverse effects of metabolic diseases, such as HUA and obesity, and improve eating habits to maintain a uric acid at normal levels.

### Study limitations

There were several limitations to this study. First, the participants in the control group included some sub-health groups. If the participants in the control group were thoroughly healthy, the effect of HUA on CAD would be more prominent. Second, most of our participants were high-risk groups of cardiovascular disease. Thus, the positive rate of coronary angiography was high. These populations may not represent the general population. Third, the effect of anti-HUA drugs on the progression of CAD should be investigated further in large randomized trials, which may potentially provide new therapeutic approaches for the prevention and treatment of CAD.

## Conclusions

In our study, HUA was shown to be significantly associated with the presence of CAD in non-smokers ≤ 35 years of age.

## Additional file

Additional file 1: **Table S1.** Interaction effect of hyperuricemia and cigarrete smoking on CAD. **Table S2.** Relationship between hyperuricemia/uric acid and smoking in the special group. **Figure S1.** Flowchart of the study. (DOCX 546 kb)

## Abbreviations

BMI: Body mass index
CAD: Coronary artery disease
CI: Confidence interval
HDL-C: High density lipoprotein cholesterol
LDL-C: Low density lipoprotein cholesterol
MS: Metabolic syndrome
NCEP ATP III: The Adult Treatment Panel III (ATP III) of the National Cholesterol Education Program
OR: Odds ratio
SUA: Serum uric acid
TC: Total cholesterol
TG: Triglyceride
