# Supplementary material for: Hyperuricemia and smoking in young adults suspected of coronary artery disease ≤ 35 years of age: a hospital-based observational study
Source: BMC Cardiovasc Disord. 2018 Aug 31;18:178. doi: 10.1186/s12872-018-0910-5 (PMC6119325; doi:10.1186/s12872-018-0910-5)
Supplement: Supplementary file 1 — Table S1. Interaction effect of hyperuricemia and cigarrete smoking on CAD. Table S2. Relationship between hyperuricemia/uric acid and smoking in the special group. Figure S1. Flowchart of the study. (DOCX 546 kb) [file 12872_2018_910_MOESM1_ESM.docx]

Table S1 Interaction effect of hyperuricemia and cigarrete smoking on CAD

| Exposure | Cigarrete smoking | Crude | Model I^*^ | Model II^**^ |
| --- | --- | --- | --- | --- |
| Hyperuricemia |  |  |  |  |
| No | No | Reference | Reference | Reference |
| Yes | No | 2.08 (1.28, 3.40) | 2.02 (1.18, 3.45) | 2.02 (1.18, 3.46) |
| No | Yes | 1.88 (1.37, 2.59) | 1.68 (1.19, 2.38) | 1.82 (1.27, 2.60) |
| Yes | Yes | 1.90 (1.33, 2.71) | 1.54 (1.03, 2.29) | 1.60 (1.06, 2.42) |
| P interaction |  | 0.0155 | 0.0105 | 0.0079 |

Abbreviations: CAD=coronary artery disease; HDL-C=high density lipoprotein cholesterol; LDL-C=low density lipoprotein cholesterol; BMI=body mass index; CI=confidence interval; OR=odds ratio.

^*^Adjusted for Low HDL-C, Hypercholesterolemia, Hypertriglyceridemia, Metabolic syndrome, BMI, Diabetes mellitus, Family history of CAD, Blood urea nitrogen, Serum creatinine.

^**^Adjusted for Age, High LDL-C, Low HDL-C, Hypercholesterolemia, Hypertriglyceridemia, Metabolic syndrome, BMI, Hypertension, Diabetes mellitus, Family history of CAD, Blood urea nitrogen, Serum creatinine and Alcohol drinking.

Table S2 Relationship between hyperuricemia/uric acid and smoking in the special group

|  |  | Non-smokers | Smokers | P value |
| --- | --- | --- | --- | --- |
| Uric acid, mg/dl | Total | 6.19 ± 1.50 | 6.71 ± 1.56 | <0.001 |
|  | Male | 6.45 ± 1.43 | 6.71 ± 1.57 | 0.009 |
|  | Female | 4.90 ± 1.10 | 6.12 ± 1.23 | 0.019 |
| Hyperuricemia, n (%) | Total | 110 (28.28) | 279 (38.54) | <0.001 |

Values are given as mean ± standard deviation or number (%).

Figure S1 Flowchart of the study.


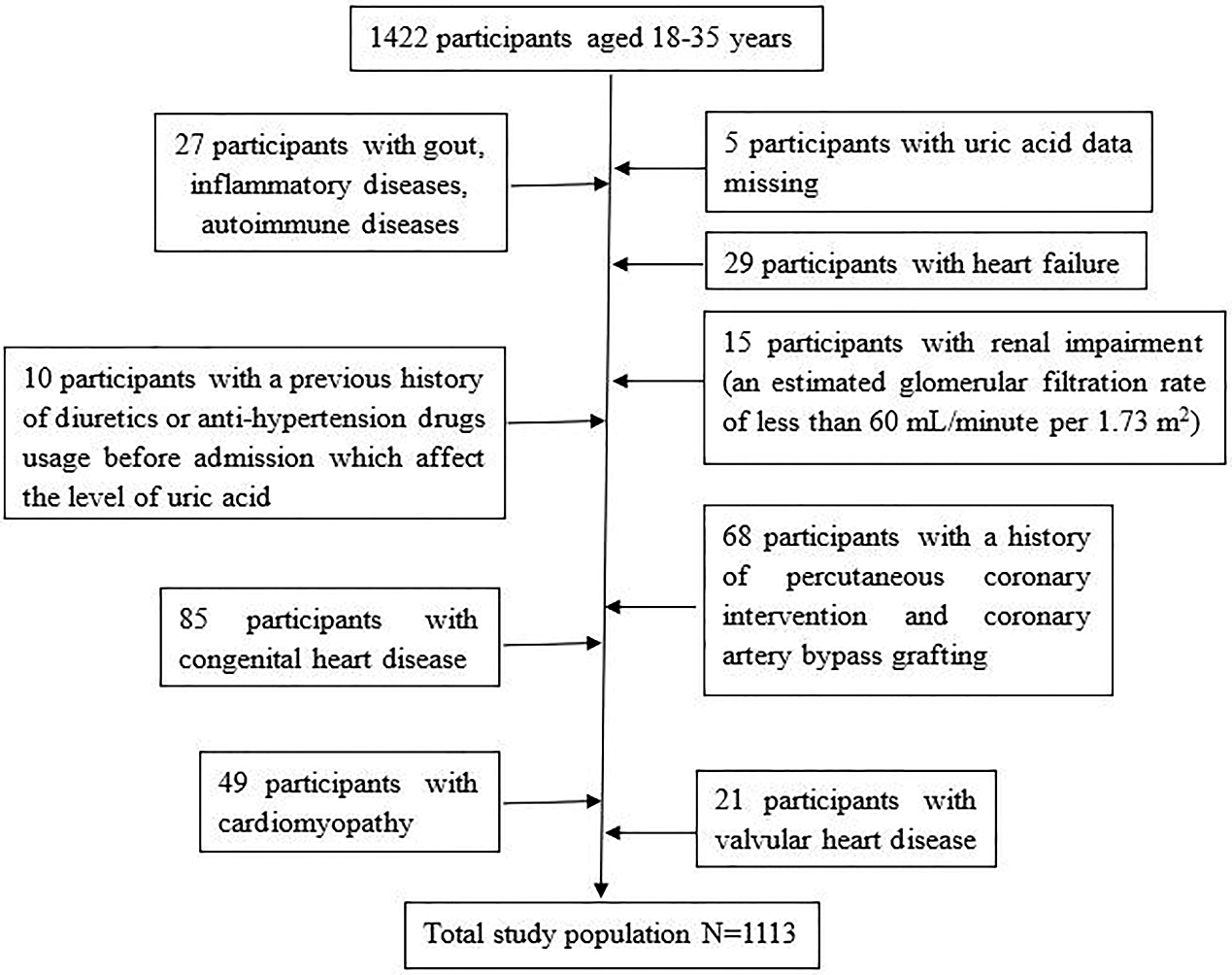


Flowchart of the study shows participants selection based on both the inclusion and exclusion criteria among young adults aged 18-35 years old who underwent coronary angiography for the first time in Anzhen Hospital. Total of 1113 participants were included in this analysis.
